# Post treatment of anaerobically treated brewery effluent using pilot scale horizontal subsurface flow constructed wetland system

**DOI:** 10.1186/s40643-020-00356-0

**Published:** 2021-01-28

**Authors:** Ermias Alayu, Seyoum Leta

**Affiliations:** 1 Center for Environmental Science, College of Natural and Computational Science, Addis Ababa University, Arat kilo campus, Post – Graduate building, 6th floor, P.O.Box 1176, Addis Ababa, Ethiopia; 2 Department of Chemistry, College of Natural and Computational Sciences, Assosa University, P.O.Box 18, Assosa, Ethiopia

**Keywords:** Horizontal subsurface flow constructed wetland, *Cyperus alternifolius*, *Typha latifolia*, Brewery wastewater, Combined macrophyte nutrient uptake potential, Tertiary treatment

## Abstract

The anaerobic process is considered to be a sustainable technology for the treatment of wastewaters rich in organic matter mainly due to its lower energy consumption and production of value-added products such as biogas and organic fertilizer. However, it cannot be seen as providing ‘complete’ environmental solution as its treated effluents would typically not meet the desired discharge limits in terms of residual carbon, nutrients and other pollutants. This has given impetus to subsequent post treatment in order to meet the environmental standards and protect the receiving water bodies and environment. The aim of this study was to evaluate the post-treatment potential of a pilot scale two-stage horizontal subsurface flow constructed wetland (HSSFCW) system planted with *Cyperus alternifolius* and *Typha latifolia*, respectively, for enhanced removal of residual carbon and nutrient from an up-flow anaerobic sludge blanket (UASB) reactor treated brewery effluent. A pilot scale two-stage HSSFCW was integrated with the UASB reactor, and its performance efficiency was assessed for the removal of total suspended solids (TSS), chemical oxygen demand (COD), total nitrogen (TN), ammonium–nitrogen (NH_4_–N), total phosphorous (TP), and orthophosphate (PO_4_^3−^). Macrophytes aboveground biomass and nutrient accumulation potential were also determined following standard methods. The results from this study showed that *Cyperus alternifolius* planted CW cell removed 68.5% TSS, 74.2% COD, 55.7% TN, 68.6% NH_4_–N, 41.1% TP and 48.1% PO_4_^3−^. Moreover, further polishing with *Typha latifolia* planted CW cell enhanced the removal efficiencies to 89% TSS, 92% COD, 83.6% TN, 92.9% NH_4_^–^N, 74.4% TP, and 79.5% PO_4_^3−^. Strong linearity and Pearson correlation was found between macrophyte biomass and nutrient accumulation in each CW cell (*Cyperus alternifolius*: *R*^2^ = 0.91, *r* = 0.97 for TN; *R*^2^ = 0.92, *r* = 0.96 for TP; and *Typha latifolia*: *R*^2^ = 0.96, *r* = 0.98 for TN and TP), and showed substantial nutrient reduction with cumulative nutrient accumulation of 1290 gTNm^−2^ and 708.7 gTPm^−2^ in the complete system. The performance of the pilot CW system as a tertiary treatment for brewery wastewater showed that the effluent meets the permissible discharge standards throughout the year excluding phosphorous. 
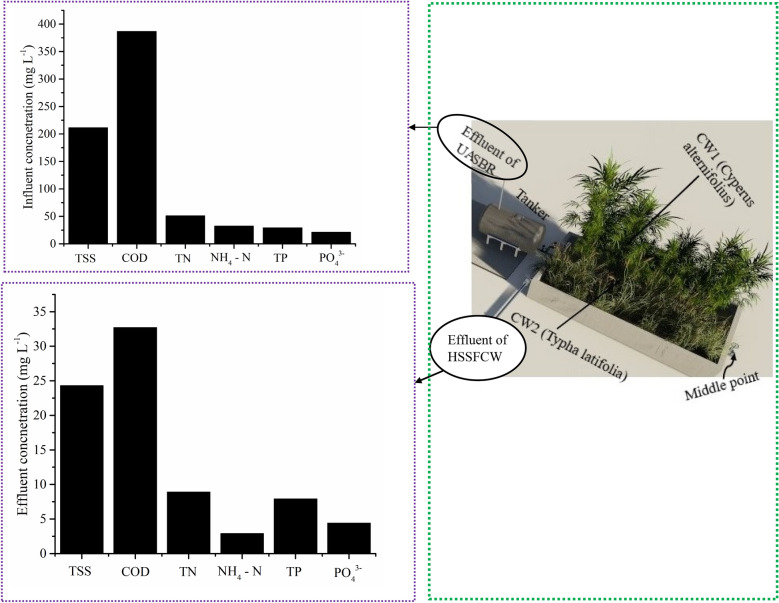

## Highlight

- Full scale UASB reactor performance has been evaluated and is effective in COD removal but very limited in nutrient removal capacity.
- The purpose of this study was to assess the post treatment potential of the series connected two–stage HSSFCW for UASB reactor treated brewery effluent.
- The two–stage HSSFCW polishing totally achieved the national discharge limit for TSS, COD, TN, NH_4_-N.
- Consideration should also be given to the use of methane instead of flaring by means of reuse.

## Introduction

In developing countries, only 8% of wastewater is treated, and reckless disposal of untreated municipal and agro-processing industrial wastes laden with organic matter, nutrients, and other hazardous chemicals into water bodies and the environment poses ecological and health risks (Nebyou 2011; Worku et al. 2018; Ashekuzzaman et al. 2020). Similarly, discharging of untreated or partially treated high-strength wastewater from brewery also poses contamination of water bodies and the environment (Hultberg and Bodin 2017; Mohan et al. 2018). Brewery industries held an enormous economic position in global beer production greater than 134 billion liters in the past, and become the 5th drinks internationally with an average annual consumption rate of 23 L per individual (Simate et al. 2011). In Ethiopia, more than 700 million liters of beer were produced per year with 24% consumption rate (Nebyou 2011). On the other hand, beer production is water demanding process, which consumes 4.5 L of water per liter of beer production (Karina et al. 2017), and produced 3 to 10 L of highly polluting effluents (Simate et al. 2011). While, Ethiopian breweries consumed on average 5.6 m^3^ liter of water per liter of beer production and generate approximately 3.9 m^3^ wastewater (Worku et al. 2018). In Ethiopia, most of the breweries drain their effluents into rivers and nearby environment, and cause water bodies to stench, discoloration, and oily nature (Belay and Sahile 2013), while effluents used for irrigation can pose soil pollution problem (Oljira et al. 2018). To reduce these problems, environmental authorities are pressuring the breweries to manage their effluents below environmental standards. Few breweries have shown to adopt treatment technologies such as an anaerobic process with the target of capturing energy. However, the final effluent contains residual organic, suspended solid and nutrient concentrations that exceed the national discharge standards (Bulla 2014). Tyagi et al. (2009) have also reported that the anaerobic treatment process was infective to eliminate residual organics, suspended solids, and nutrient to the level of meeting discharge limits. It is evident that anaerobic pretreatment is a known desirable scenario for the robust removal of organic matter from various types of wastewaters (Caliskan et al. 2014), and reduce methane emissions by converting organic matter into value-added products (Karina et al. 2017). Using the aerobic process as a polishing system for the removal of residual organic and nutrient is an energy and chemical-intensive process; requires high operational costs and expensive computerized treatment units; generate secondary pollutants, and its expertise personnel requirement couldn`t be affordable for developing countries (Badejo et al. 2014). Even though several other alternative polishing options are available, naturally working constructed wetlands (CW) are gained popularity as an ecofriendly polishing technology, and recently utilized for different anaerobic reactor effluent treatment (Zeb et al. 2013; Jamshidi et al. 2014).

Moreover, the application of CW integrating with an anaerobic treatment system has a more significant benefit for resource-scarce countries to manage wastewaters with other multiple advantages (El-Khateeb and El-Bahrawy 2013). The anaerobic pretreatment reduce the CW area requirement by 30 to 60% (Alvarez et al. 2008), avoid chemical or energy requirements (Zeb et al. 2013; Jamshidi et al. 2014), reduce effluent hydraulic retention time (HRT), escaping CW clogging problem, increase the durability of CW (Ayaz et al. 2015), and perform robust organic compound removal through a stepwise microbial degradation process such as hydrolysis, acidogenesis, acetogenesis and methanogenesis into methane (CH_4_), carbon dioxide (CO_2_), and other trace gases (Menzel et al. 2020). While the CW polishing mitigates a wide variety of residual pollutants such as organic matter, suspended solids, metals, nutrient, and pathogens (Sedaqua 2013), through a variety of physical and biochemical processes (Vymazal 2007). Very few single-stage HSSFCWs were investigated for various types of wastewater post-treatment with promising pollutant reductions (de la Varga et al. 2013; Zeb et al. 2013). However, the treatment of high strength food processing wastewaters using this polishing stage is difficult to produce high-quality effluent (Vymazal 2005). Studies recommended a series of interconnected HSSFCW polishing system for enhanced removal of pollutants and discharging high quality effluents (Morino-Solis et al. 2015; Cheng et al. 2010). Studies have also indicated that *C. alternifolius* and *T. latifolia* individual-based wastewater treatment showed good removal efficiencies for organic matter and nutrient. For instance, *C. alternifolius* planted HSSFCW removed 95% COD and 93% TSS (Sa’at et al. 2017); while *T. latifolia* removed 92% TSS, and 79% COD (Ciria et al. 2005). Terfie and Asfaw (2015) reported up to 82% NH_4_-N removal efficiency from tannery wastewater using *C. alternifolius*, and Gebeyehu et al. (2018) reported up to 80% TN, 65% NH_4_–N, and 70% PO_4_^3−^ removal efficiencies from brewery wastewater using *T. latifolia.* However, there is lack of concrete and reliable scientific data on their combined performance for treating high strength wastewaters such as food processing industries. Studies suggested that use of combined macrophytes in the series improves pollutant removals (Rezaie and Salehzadeh 2014) through increasing biomass production, augmenting oxygen availability, microbial activity, and nutrient uptake (Geng et al. 2017).

However, the performance of a given CW system will largely be influenced by local specific environmental conditions, system design and plant types, among others. Thus, implementing CW for a given wastewater type and local environmental conditions requires local pilot investigations to assess the performance of HSSFCWs planted with different plant species grown in a given environmental conditions. *C. alternifolius* and *T. latifolia* have different growth rates and root structures, and these make for interesting comparisons of the performance of these two plant species in series connected HSSFCWs. Detailed research data on the efficiency of CWs, performance and appropriate set-up are still missing for brewery wastewater treatment. However, efforts have already been made selecting efficient macrophyte species to this particular wastewater (Kenatu 2011; Gebeyehu et al. 2018; Badejo et al. 2014). In addition, a system with a combination of UASB reactor and two-stage HSSFCWs has not yet been applied to brewery wastewater treatment. To generate empirical information to the operational condition of CWs, we developed a series connected two-stage HSSFCW system, one planted with *C. alternifolius* and another planted with *T. latifolia*, for the enhanced removal of organics and nutrients from anaerobically treated brewery effluent.

## Materials and methods

### Experimental location

A horizontal subsurface flow constructed wetland (HSSFCW) pilot plant was built on the premises of Kombolcha Brewery connected with the existing an up-flow anaerobic sludge blanket (UASB) treatment plant in Kombolcha town, Northern Ethiopia, located at 11^°^04´42.43´´N 39^°^43´34.45´´ E and 1833 m above sea level, an area with annual average minimum and maximum temperatures varying between 6.1–15.2 °C and 24.7–30.4 °C, respectively, and mean annual rainfall of 255.7 mm.

### Experimental design and setup

Biological oxygen demand (BOD) is the basis for determining the size of the wetland area required using a first order plug flow model equation proposed by Kickuth, and is commonly used for sizing of HSSFCW system for domestic sewage wastewater treatment (UN-HABITAT 2008). The size of each series connected pilot scale HSSFCW was determined using the daily hydraulic flow rate, Q_d_ (0.698 m^3^d^−1^), influent BOD concentration (223.9 mgL^−1^), the recommended national discharge standard limit of BOD (60 mgL^−1^), and BOD rate constant (K_BOD_). The K_BOD_ is usually lower, varied in between 0.07–0.1 md^−1^. But, according to Vymazal and Kropfelova (2008), many countries used 0.08 md^−1^. The effective aspect ratio (L/W) of each cell was 5:1, which is in agreement with the recommended value of 5:1 (Kadlec and Wallace 2009). The theoretical hydraulic retention time (HRT) was 4 days, which is estimated by Eq. (2) using the average flow through the system (0.698 m^3^d^−1^), the dimension of each series connected cells (7.56 m × 1.52 m), the operating water level (0.45 m), and the initial (clean) porosity of the media (0.27), which was experimentally determined. The hydraulic loading rate (HLR) (md^−1^) is the volume of wastewater loaded per unit surface area of CW, calculated by Eq. (3):

1 $$A_{s} = \frac{{Q_{d} \left( {{\text{In}}^{{C_{i} }} - {\text{In}}^{{C_{e} }} } \right)}}{{K_{{{\text{BOD}}}} }}$$

2 $${\text{HRT }}\left( {{\text{day}}} \right){ = }\frac{{{\text{LWDP}}}}{{\text{Q}}}$$

3 $${\text{HLR = }}\frac{{\text{Q}}}{{{\text{A}}_{{\text{s}}} }}$$

where *Q* (m^3^d^−1^) is the hydraulic flow rate, *C*_e_ and *C*_i_ are the effluent and influent concentrations, *L* is length of wetland (m), *W* is width of wetland (m), As (m^2^) is the surface area of the HSSFCW unit, d (m) is the influent flow depth, and p is the porosity (%) of the media used.

**Fig. 1:**
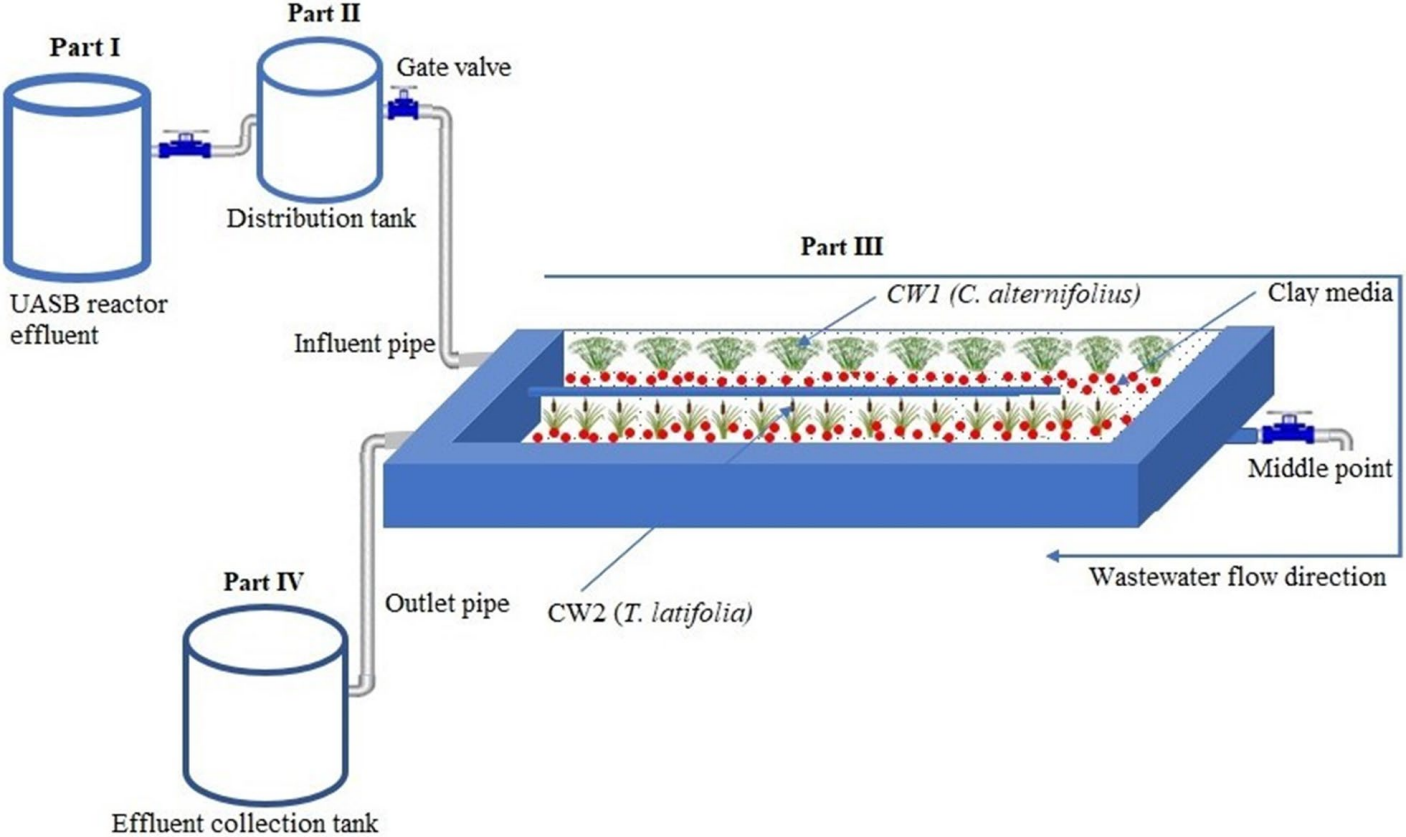
Schematic diagram of the experimental setup of the horizontal subsurface flow constructed wetland

**Fig. 2:**
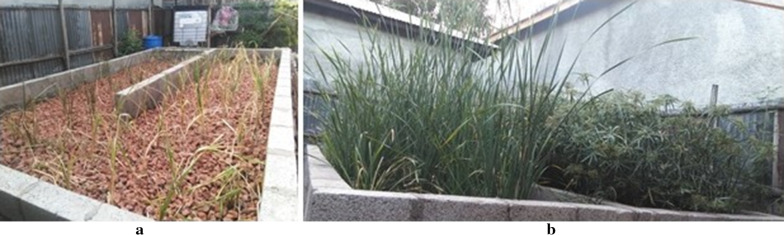
The pilot scale two-stage HSSFCWs planted with *C. alternifolius* and *T. latifolia* at the start of operation (**a**) and fully grown plants during the experimentation (**b**)

The experimental setup is shown in Fig. 1 consists of four parts: UASB reactor treatment plant -existing (I); distribution tank (II), two stage HSSFCWs (III) and collection tank (IV). The UASB reactor, distribution tank and the two HSSFCWs were connected by a PVC pipe with control valves. The volume of the existing UASB reactor is 592 m^3^, which works based on the average hydraulic flow rate of 840 m^3^ per day. The UASB reactor pre-treatment are consisted of screens, and buffering tank. The screens were used to remove oil and grease, heavy solid materials. The buffering tank was used to balance the pH variation and flow from operation of the brewing process. One 3000 L volume distribution tank was used as storage tanks from the UASB reactor plant. The two equally sized series connected wetland cells had L × W × D dimensions of 7.56 m × 1.52 m × 0.45 m. The HSSFCW body was made from concretes and well smoothed to avoid any seepage. The outlet pipe was installed 0.35 m above the floor inside the HSSFCWs and was connected to the collection tank using T- fitting pipe. The series connected HSSFCWs body was made from concretes and the interior region was well insulated. A 15–25 mm size clay rock media composed of 76.36% w/w SiO_2_, 13.69% w/w Al_2_O_3_, 4.24% w/w Fe_2_O_3_, 1.52% w/w CaO, < 0.1% w/w MgO; obtained from Mitikolo was filled to the depth of 0.45 m. Two locally available macrophytes were collected from Borkena River and identified at Addis Ababa University National Herbarium, and planted in the two-stage HSSFCW unit without mixing orderly in the first and second cells in August 2018. As indicated in Fig. 2ab, the plantation order was preceded by *C. alternifolius* due to its high pH resistance (Miyazaki et al. 2004), high productivity, relatively strong root system, easy adaptation to organic load changes, salinity tolerance, and high nutrient absorption capacity (Bilgin et al. 2014), followed by *T. latifolia* due to its short root length (Bonanno and Cirelli 2017), active carbon-producing potential around the rhizosphere for biological activities (Fahlgren 2017), less salinity tolerance, and ability to mitigate nutrient-rich wastewater (Mollard et al. 2013). In addition, these macrophytes biomass use for making floors, animal feed, making roofs, and mattresses (Assefa et al. 2013) and low evapotranspiration rates (Leto et al. 2013) being considered as a selection criterion. The endorsed macrophytes were acclimatized with diluted wastewater (75:25; brewery effluent to clean pipe water ratio) from a reservoir continuously.

### Operation of the HSSFCWs

The operation of the experiment was initiated in January 2018 by pumping a predetermined daily hydraulic flow rate of 0.698 m^3^d^−1^ through controlling it by a 2-inch gate valve and the performance study was monitored for 1 year, until December 2019. The series connected two-stage HSSFCWs was continuously fed with anaerobically treated effluent from the distribution tank, controlled with the help of a gate valve using a stopwatch and a measuring cylinder at the inlet of the CW. The water depth was maintained at 0.45 m within the wetland with the aid of fixed outlet pipes. The study was conducted at a HLR of 0.03 md^−1^ with a corresponding hydraulic retention time of 4 days.

### Wastewater sampling and quality analysis

Wastewater samples were collected three times per month from the inlet and outlet of UASB reactor treatment plant, or inlet of CW1, the middle point between the two macrophytes, and outlet of CW2 for 1 year using a pre-cleaned ethylene polyvinyl bottles and transported to the laboratory for immediate analysis. On-site wastewater parameters such as pH, temperature, and dissolved oxygen (DO) were measured using a handheld IntelliCAL™ pH/temperature digital probe (HACH® HD30d Flexi, Loveland, USA), and DO meter (YSI 550A, Yellow Springs, OH, USA), respectively. Whereas, the laboratory analyses for parameters such as TSS (oven-dry method), COD (reactor digestion method), TN (persulfate digestion method), NH_4_–N (Nessler Method), TP (Molybdovanadate with Acid persulfate Digestion Method), and PO_4_^3−^ (PhosVer 3® Method) were measured using a spectrophotometer (DR/890 HACH, Loveland, USA) according to HACH instructions (APHA 1998). The pollutant removal efficiency (RE) and hydraulic loading rate (HLR) were computed following Eqs. (4) and (5) (Juang and Chen 2007):

4 $${\text{RE}}\left( {\text{\% }} \right) = \frac{{{\text{C}}_{{\text{i}}} - {\text{C}}_{{\text{e}}} }}{{{\text{C}}_{{\text{i}}} }} \times 100$$

5 $$\user2{ }{\text{MLR }}\left( {{\text{gm}}^{{ - 2}} {\text{d}}^{{ - 1}} } \right){\text{ = C}}_{{\text{i}}} {\text{ x HLR}}$$

### Macrophytes biomass and nutrient content measurement

*C. alternifolius* and *T. latifolia* aboveground biomass (AGB) were collected every three months from the HSSFCW cells for 1 year and transferred to the laboratory using plastic bags for dry weight biomass and nutrient content determination. Macrophyte AGB parts were oven-dried at 105 °C for 24 h through extending the time until a constant weight was achieved, and weighed (Maqbool and Khan 2013). Dried AGB parts was fine grounded to < 2 mm sieve. Then, TN was determined using potassium-persulfate decomposition and UV–Vis spectrometry method (APHA 1998). Whereas, TP was determined by digesting 0.5 g samples in aquaregia for 2hrs at 90 °C on a hot plate and determined using Inductively Coupled Plasma (ICP-OES, Arcos spectrophotometer, Germany). The nutrient accumulation (N) in the macrophytes AGB was estimated following Eq. (6).

6 $${{\text{N}} ({\text{gm}}^{-2}) = {\text{DM}}}_{{{\text{macrophyte}}}} \times {\text{C}}_{{{\text{macrophyte}}}}$$

where DM is the dry weight biomass of macrophytes (kgm^−2^); *C* is the concentration of TN (gN kg^−1^) and TP (gPkg^−1^) in the macrophytes.

### Data analysis

The data obtained from the study were analyzed using descriptive and inferential statistical data analysis using Microsoft excel, 2013 and OriginPro2017. The results were expressed in terms of mean and standard deviation values. Results were presented using graphs and tables.

## Results and discussion

### Treatment potential of UASB reactor

Evaluation of treatment performance full scale UASB reactor for Kombolcha brewery effluent showed 58.6% TSS and 79.3% COD removal efficiencies at 11 h HRT with 1170.1 Nm^3^d^−1^CH_4_ production. This study result is somewhat higher than reported result of 58%TSS and 41% COD removals at 11 h HRT (Alvarez et al. (2006), and Khan et al. (2014) observed average UASB reactor efficiency of 75% of TSS and COD removals operated at 8 h HRT. With regard to nutrient removal, the UASB reactor performance evaluation showed removal efficiencies of 34.4% TN, 32.2% TP, and 38.9% PO_4_^3^ (Table 1), which are exceeded from Torres and Foresti (2001) reported results of 10–25% TKN and 10–20% PO_4_^3−^, and El–Khateeb and El–Bahrawy (2013) reported average removal of 11.3% TKN and 23% TP.

**Table 1:** UASB reactor influent and effluent concentrations (mgL^−1^) and percentage removal efficiency in brackets

Month	UASB influent concentration (mean ± SD)					UASB effluent concentration (mean ± SD)				
	TSS	COD	TN	TP	PO_4_^3−^	TSS	COD	TN	TP	PO_4_^3−^
Jan	652 ± 3	2866 ± 20.8	122 ± 3	46.9 ± 1.2	36.9 ± 1.5	274.4 ± 12 (57.9)	614 ± 5.3 (78.6)	101.4 ± 3 (16.9)	32.5 ± 2.4 (30.7)	25 ± 1.4 (32.2)
Feb	642 ± 1.5	2663.3 ± 58.6	102.7 ± 1.5	45.3 ± 3.2	32.4 ± 1.1	263.7 ± 5.4 (58.9)	545.7 ± 10 (79.5)	77.4 ± 2.2 (24.1)	28.4 ± 1 (37.3)	20.6 ± 0.4 (36.4)
Mar	492.7 ± 2.3	1210 ± 30	84.9 ± 2.3	39.4 ± 3	24.8 ± 1	180.2 ± 1.2 (63.4)	338.7 ± 10 (72)	43.6 ± 3.2 (48.6)	25.4 ± 2.4 (34.5)	17.3 ± 0.9 (30.2)
Apr	552.7 ± 3.2	2260 ± 43.6	60.3 ± 3.2	44.8 ± 1.4	37.7 ± 2.8	256 ± 2 (53.7)	443.7 ± 5.9 (80.4)	38 ± 1.2(37)	33.4 ± 2.9 (25.4)	27.5 ± 1.2 (27.1)
May	437.7 ± 7.4	1056.7 ± 35.1	84.3 ± 7.4	58.4 ± 2.2	51.4 ± 5.1	210.9 ± 6.8 (51.8)	278.2 ± 18 (73.7)	57 ± 3.9(32.4)	42.9 ± 2.9 (26.4)	29.9 ± 1.6 (41.8)
Jun	530.3 ± 6	1884.3 ± 32	85.3 ± 6	41 ± 2	37.1 ± 1.2	212 ± 12 (60)	350.2 ± 16 (81.4)	63.3 ± 2.1(25.8)	27.7 ± 2.1 (32.4)	20.1 ± 1.6 (45.8)
Jul	576.7 ± 2	1432.7 ± 31.9	58 ± 2	44.8 ± 0.2	33 ± 0.3	184.3 ± 5.1 (68)	207.3 ± 3.1 (85.5)	29.4 ± 1(49.3)	24.4 ± 3.4 (45.5)	14.4 ± 1 (56.4)
Aug	452 ± 3.2	1915.7 ± 38.8	50.3 ± 3.2	29.7 ± 0.7	24.1 ± 1.2	123.7 ± 5.1 (72.6)	334.5 ± 0.5 (82.5)	25 ± 1.9(50.3)	17.9 ± 3.1 (39.7)	10.2 ± 0.3 (57.7)
Sept	567.3 ± 3.5	1360 ± 32.4	62.7 ± 3.5	60.1 ± 1	50.6 ± 1.2	239.3 ± 6.1 (57.8)	383.3 ± 9.9 (71.8)	32.8 ± 1.8 (47.7)	41.7 ± 2 (30.6)	32.9 ± 1.4 (35)
Oct	455.7 ± 3.2	2376.7 ± 41.6	71.7 ± 3.2	33.9 ± 1.6	26.9 ± 1.1	211.4 ± 2.1 (53.6)	436 ± 2 (81.7)	48.6 ± 7.2(32.2)	23.2 ± 1 (31.6)	19.6 ± 1.3 (27.1)
Nov	366 ± 4.9	1669 ± 3	64.3 ± 4.9	28.4 ± 1.1	21.9 ± 1.7	177.4 ± 12 (51.4)	313 ± 2.6 (81.2)	34.9 ± 4.6(45.7)	20.3 ± 2.2 (28.5)	12.6 ± 0.3 (41.1)
Dec	393.3 ± 2.7	1750 ± 18.7	88.3 ± 2.7	41.3 ± 2.6	32.5 ± 2.4	200.9 ± 7.4 (48.9)	392.7 ± 3.5 (77.6)	61.3 ± 2.1(30.6)	29.4 ± 1.6 (28.8)	22.6 ± 1.3 (31.1)
*p*-value	< 0.05	< 0.05	< 0.05	< 0.05	< 0.05	< 0.05	< 0.05	< 0.05	< 0.05	< 0.05

Many studies reported that UASB reactors are limited in removal of nutrients (Cheng et al. 2010; El–Khateeb and El–Bahrawy 2013). The nutrient removal drawback of UASB reactor is associated with mineralization or hydrolysis phenomenon, which increases nutrient concentrations in the anaerobic reactors particularly into ammonia and orthophosphate (Moawad et al. 2009). Overall, characterization of the present UASB reactor brewery effluent suspended solids, and organic matter contents showed substantial fluctuations and exceeded the national discharge standard limit (EEPA 2003). Many research evidences showed that treatment effectiveness of UASB reactor is influenced by several factors such as nature of suspended solids, reactor temperature, organic loading rate, hydraulic retention time, feeding mode or up flow velocity, amount of seed sludge on reactor start up (Hu 2013; Torretta et al. 2017). Anaerobic reactor pollutant abatement efficiency is mainly affected by temperature and pH. Normally, microorganism activity in the anaerobic reactors widely performed at the mesophilic range (i.e., 25–38 °C) pH range of 6.8–7.2 (Saleh and Mahmood 2004). The present UASB reactor influent temperature varied in between 30.6–35.8 °C with an average value of 34 ± 1.6 °C, which meets the above mesophilic range and may enhance microbial activities. On contrary, too lower psychrophilic and higher thermophilic conditions cease the growth and activity of methanogens (Rizvi et al. 2015). Whereas, the pH value was fluctuated between 6.3 and 9.07, with mean value of 7.2 ± 0.8, may be due to the use of nitrogen and phosphorous-containing sanitizing chemical agents such as caustic soda, nitric acid, phosphoric acid, etc., and high content of nutrients derived from malts, and yeast cells (Gebeyehu et al. 2018; Amenorfenyo et al. 2019).

### Post-treatment potential of two-stage HSSFCWs

#### Characteristics of UASB effluent temperature, pH, and DO

**Fig. 3:**
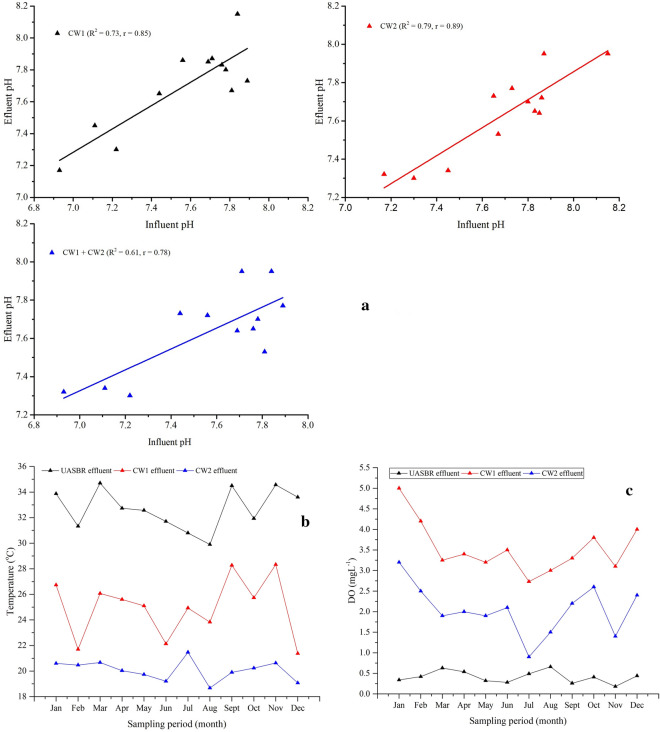
**a** Correlation of inlet and outlet pH; variation of influent and effluent (**b**) temperature, and (**c**) DO in the series-connected two-stage HSSFCWs

The UASB reactor effluent average pH value was varied from 6.9 to 7.9 with an average value of 7.6 ± 0.3. But, after polishing with the series connected two-stage HSSFCWs, its value was increased to 7.7 ± 0.3 (7.2–8.1) in CW1 and decreased to 7.6 ± 0.2 (7.3–7.9) in CW2. In agreement to this study, Merino-Solís et al. (2015) observed pH value variation in the HSSFCW treatment stages during the treatment of anaerobic reactor effluent. In contradict, Raboni et al. (2014) observed pH reduction in the HSSFCWs during the treatment of the UASB reactor effluent domestic wastewater. However, studies indicated that *C. alternifolius* and *T. latifolia* based treatment of domestic wastewater neutralizes wastewater pH value close to 7.0 (Neralla et al. 2010). But, in this study, moderate relationship was found between the inlet and outlet pH values across the stages and complete system (*R*^2^ = 0.8, *r* = 0.85 for CW1; *R*^2^ = 0.83, *r* = 0.89 for CW2; and *R*^2^ = 0.75, *r* = 0.78 for CW1 + CW2) throughout the study periods (Fig. 3a), may be due to a certain pH calibration with 30%HCl and 50%NaOH in the UASB reactor pretreatment stage for the proper functioning of microorganisms in the anaerobic reactor. In CW nitrifying and denitrifying bacteria´s activity is influenced by pH, and affects its nitrogen removal mechanism. For instance, pH > 8.0, decrease the nitrifying and denitrifying bacteria’s activity of the CW bioreactor. Unlike, in this study, the pH value meet the optimum pH ranges of 6.5 to 8.5, which is safe for both microbial activity and macrophytes growth (Vymazal 2007).

Temperature is another factor that affects organic matter and nitrogen removal mechanisms in the CW biological reactor when deviates from the favorable range of 19–34 °C. These ranges are suitable for both plant growth and microorganisms growth and activity (Zamora et al. 2019). Hence, measurement of this parameter in the UASB effluent showed a mean value of 32.7 ± 1.6 °C (29.9–34.7 °C), which meet the above normal temperature range. Treatment of UASB effluent temperature with two-stage HSSFCWs significantly reduced its value into 25 ± 2.2 °C (21.4–28.3 °C) and 20.1 ± 0.6 °C (18.7–21.5 °C), respectively, for CW1 and CW2 (Fig. 3b), and concluded that the HSSFCW treatment system acts as a buffering step. Measurement of the DO level in the UASB effluent was limited, which fluctuated from 0.18–0.63 mgL^−1^ with an average value of 0.4 ± 0.1 mgL^−1^. However, when it was treated with two-stage HSSFCWs, the influent DO concentration significant increased to 3.5 ± 0.6 mgL^−1^ (2.7–5 mgL^−1^) at the outlet of CW1. But, further polishing with CW2 decreased the CW1 DO concentration to 2.3 ± 0.6 mgL^−1^ (0.9–3.2 mgL^−1^) followed by CW2 (Fig. 3c), may be due to the difference of macrophytes aeration ability. Overall, the present results revealed that presence of macrophytes can increase the amount of oxygen transfer into the HSSFCWs. In agreement to this study, Zamora et al. (2019) were also observed a significant DO concentration level increment at the outlet of HSSFCWs. Many studies reported that the amount of DO level in the HSSFCWs may be affected by climatic conditions, loading rates, macrophytes eco-physiological and morphological features such as age, biomass, length, diameter, and porosity (Li et al. 2011; Dong et al. 2016). Besides, larger biomass of macrophytes influences the release of oxygen (Angassa et al. 2019). Similarly, the greater oxygen availability found in the present two-stage HSSFCWs may be due to the combined oxygen transferring ability and biomass of macrophytes, which agreed with La Bella et al. (2016) reported result of liable oxygen transport by *C. alternifolius* and *T. latifolia* aeration abilities. Emergent macrophytes such as C. alternifolius and T. latifolia have a broad lacunar system, which comprises of 60% of the tissues are helping in extensive oxygen transport to the rhizosphere (Rehman et al 2017).

### The pollutant removal efficiency of the two-stage HSSFCWs

#### TSS and COD removal

**Fig. 4:**
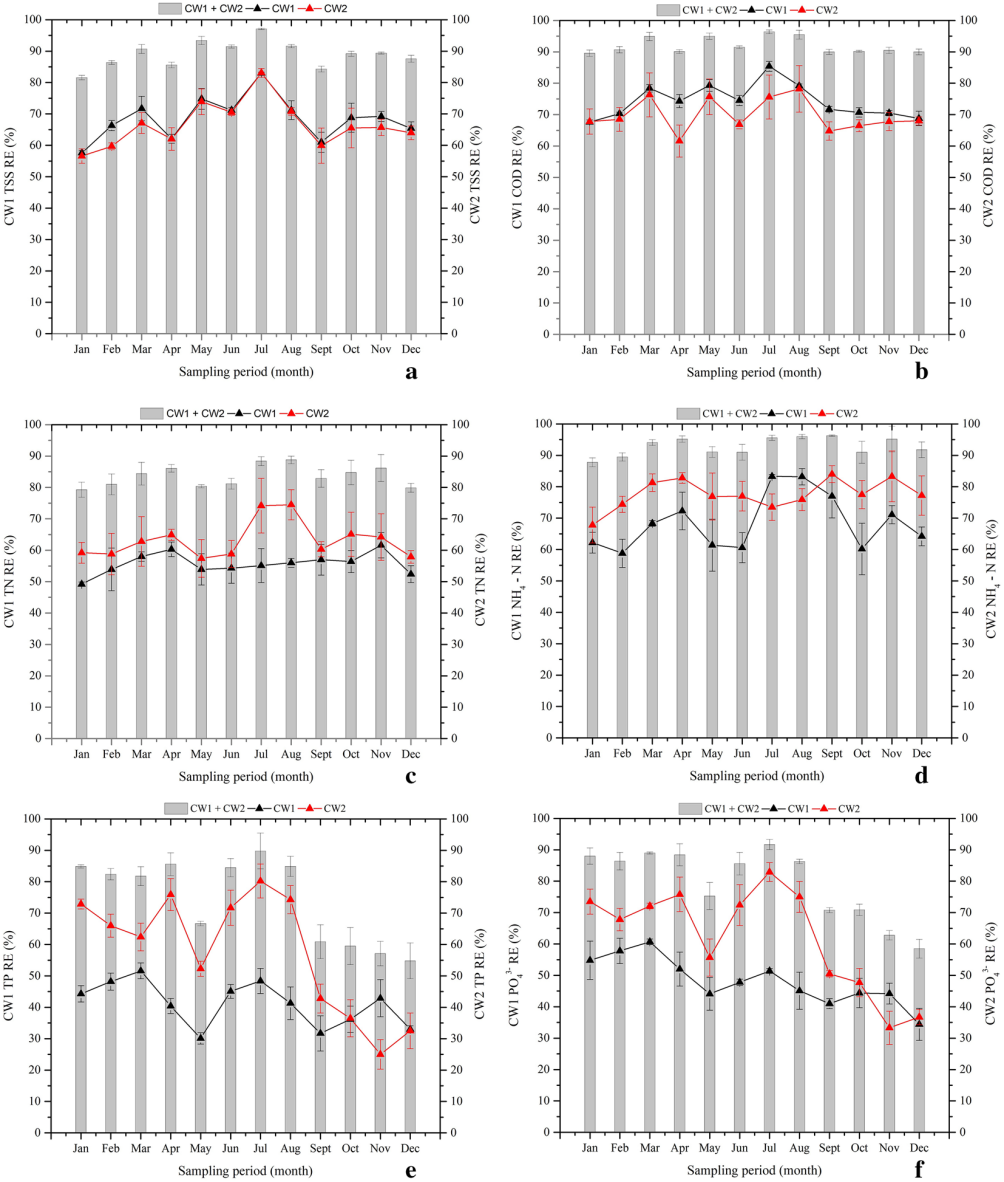
Removal efficiency variations of (**a**) TSS, (**b**) COD, (**c**) TN, (**d**) NH_4_^–^N, (**e**) TP and (**f**) PO_4_^3−^ in the two–stage HSSFCWs

**Fig. 5:**
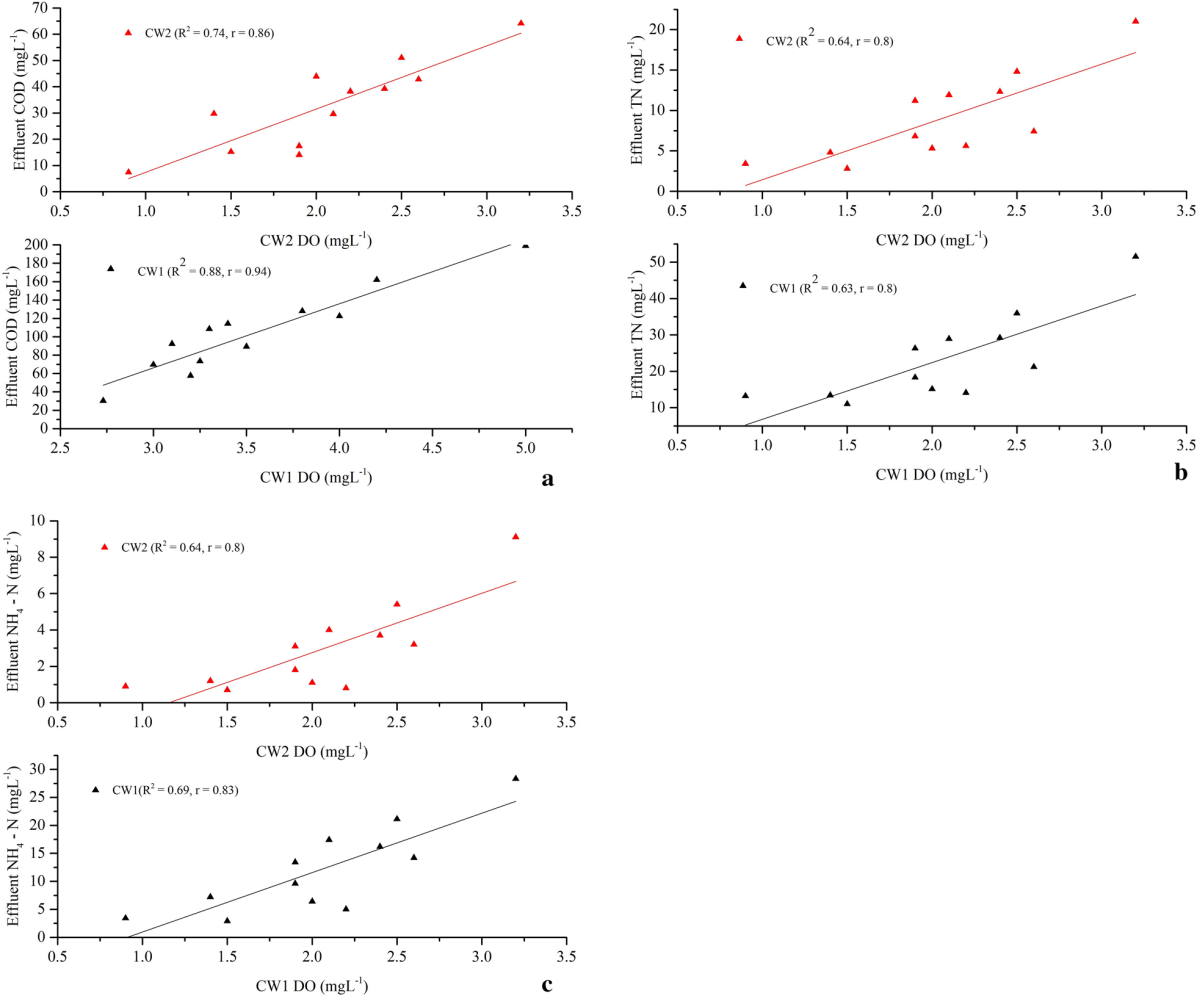
Correlation of effluent DO with (**a**) COD, (**b**) TN and (**c**) NH_4_–N effluent concentrations

Results from the 1-year operation of the two-stage HSSFCWs revealed high levels of TSS and COD reductions. As displayed in Fig. 4ab and Table 2, the limited removal of UASB reactor TSS and COD pollutants could be compensated by high efficiency in the two-stage HSSFCWs with high-quality effluent that meet the national discharge standard limit. Treatment with CW1 showed an average removal efficiency of 68.5 ± 6.5% and 74.2 ± 5.3%, respectively, for TSS and COD. While further polishing with CW2 removed 66.6 ± 7%TSS and 69.8 ± 5.3%COD. Enhanced TSS and COD removals were achieved by the complete system throughout the study period (Fig. 4ab). Carballeira et al. (2016) reported that macrophytes have an important role in TSS and COD removals. Likewise, the two macrophytes used in this study contributed favorable TSS and COD removals across the stages. But, better and almost steadier reductions were achieved by the complete system. During the UASB effluent passage from the inlet to the outlet, the effluent will come in contact with a network of aerobic, anoxic and anaerobic zones around the roots and rhizosphere of the CW macrophytes that leak oxygen to the media, and cleaned pollutants by the synergies of the physical, chemical, and biological processes in the CWs (UN–HABITAT 2008). Since macrophytes root mat enhances more solid particles adhering, filtration, and sedimentation; and organic matters biodegradation, and consumption by attached anaerobic–aerobic bacteria’s (Theophile et al. 2011; Aziz et al. 2015). On the other hand, Panwar and Makvana (2017) found that an increased DO in the effluent leads to greater purity due to the removal of pollutants, which more likely linked to the present study moderate linearity and Pearson correlations found between effluent DO and COD concentrations (*R*^2^ = 0.88, *r* = 0.94 for CW1, and *R*^2^ = 0.75, *r* = 0.86 for CW2) (Fig. 5a). Macrophytes root oxygen secretion have a positive effect on pollutant removals in a CWs (Wang et al. 2018) by providing better DO for enhanced aerobic microorganism metabolic activity that reduce organic matters through degradation (Wijaya et al. 2016).

**Table 2:** Two-stage HSSFCW system influent and effluent concentrations (mgL^−1^) and percentage removal efficiencies shown in brackets

Month	UASB reactor effluent concentration (mean ± SD)						CW1 effluent concentration (mean ± SD)					
	TSS	COD	TN	NH_4_^–^N	TP	PO_4_^3−^	TSS	COD	TN	NH_4_^–^N	TP	PO_4_^3−^
Jan	274.4 ± 12	614 ± 5.3	101.4 ± 3	74.8 ± 2.2	32.5 ± 2.4	25 ± 1.4	116.4 ± 6 (57.6)	199 ± 3 (67.6)	51.5 ± 5.6 (49.2)	28.3 ± 2.5 (62.2)	18.1 ± 1.1 (44.3)	11.3 ± 2.4 (54.8)
Feb	263.7 ± 5.4	545.7 ± 10	77.4 ± 2.2	51.2 ± 1.8	28.4 ± 1	20.6 ± 0.4	88.8 ± 3.9 (66.3)	161.9 ± 5.8 (70.3)	35.9 ± 6.3 (53.9)	21.1 ± 2.2 (58.8)	14.7 ± 0.4 (48.2)	8.7 ± 0.8 (57.8)
Mar	180.2 ± 1.2	338.7 ± 10	43.6 ± 3.2	30.3 ± 0.8	25.4 ± 2.4	17.3 ± 0.9	51 ± 7.1 (71.7)	73.3 ± 6 (78.4)	18.3 ± 1.3 (58)	9.6 ± 0.5 (68.3)	12.5 ± 1.2 (51.6)	6.8 ± 0.2 (60.7)
Apr	256 ± 2	443.7 ± 5.9	38 ± 1.2	23.1 ± 2.4	33.4 ± 2.9	27.5 ± 1.2	97 ± 4.2 (62.1)	114.2 ± 7.5 (74.3)	15.1 ± 1.3 (60.3)	6.4 ± 1.2 (72.3)	19.9 ± 2.1 (40.4)	13.2 ± 1.5 (52)
May	210.9 ± 6.8	278.2 ± 18	57 ± 3.9	34.7 ± 2.5	42.9 ± 2.9	29.9 ± 1.6	53.2 ± 5.4 (74.8)	57.6 ± 8.4 (79.3)	26.3 ± 1.1 (53.9)	13.4 ± 2 (61.4)	30 ± 1.7 (30.1)	16.7 ± 2.4(44.1)
Jun	212 ± 12	350.2 ± 16	63.3 ± 2.1	44.2 ± 2.3	27.7 ± 2.1	20.1 ± 1.6	61 ± 3.4 (71.2)	89.3 ± 2.4 (74.5)	28.9 ± 2.1 (54.3)	17.4 ± 3.1 (60.6)	15.2 ± 1.7 (45.1)	10.5 ± 0.7 (47.8)
Jul	184.3 ± 5.1	207.3 ± 3.1	29.4 ± 1	20.3 ± 1.6	24.4 ± 3.4	14.4 ± 1	31.3 ± 1.1 (83)	30.3 ± 3.7 (85.4)	13.2 ± 4.5 (55.1)	3.4 ± 0.1(83.3)	12.6 ± 0.9(48.4)	7 ± 0.4 (51.8)
Aug	123.7 ± 5.1	334.5 ± 0.5	25 ± 1.9	17.3 ± 1.6	17.9 ± 3.1	10.2 ± 0.3	35.6 ± 2.7 (71.2)	69.6 ± 2.2 (79.2)	11 ± 1.1 (56)	2.9 ± 0.2 (83.2)	10.5 ± 1 (41.3)	5.6 ± 0.9 (45.1)
Sept	239.3 ± 6.1	383.3 ± 9.9	32.8 ± 1.8	21.7 ± 2	41.7 ± 2	32.9 ± 1.4	93.4 ± 5.8 (61)	108.4 ± 2.6 (71.7)	14.1 ± 2.1 (57)	5 ± 1.1(77)	28.5 ± 5(31.7)	19.4 ± 0.9 (41)
Oct	211.4 ± 2.1	436 ± 2	48.6 ± 7.2	35.7 ± 3	23.2 ± 1	19.6 ± 1.3	66 ± 9 (68.8)	127.4 ± 7.2 (70.7)	21.2 ± 4.2 (56.4)	14.2 ± 1.8 (60.2)	14.8 ± 0.8 (36.2)	10.9 ± 1.3 (44.4)
Nov	177.4 ± 12	313 ± 2.6	34.9 ± 4.6	24.9 ± 3.9	20.3 ± 2.2	12.6 ± 0.3	34.8 ± 2.5 (69.2)	92.3 ± 3.3 (70.5)	13.4 ± 0.6 (61.6)	7.2 ± 1.1 (71.1)	11.6 ± 0.4 (42.9)	7.2 ± 0.5 (44.2)
Dec	200.9 ± 7.4	392.7 ± 3.5	61.3 ± 2.1	45.3 ± 4.5	29.4 ± 1.6	22.6 ± 1.3	69.5 ± 1.7 (65.4)	122.4 ± 9.1 (68.8)	29.2 ± 2.5 (52.4)	16.2 ± 2.5 (64.2)	19.7 ± 0.8 (33)	14.7 ± 1.3 (34.4)
*p*-value	< 0.05	< 0.05	< 0.05	< 0.05	< 0.05	< 0.05	< 0.05	< 0.05	< 0.05	< 0.05	< 0.05	< 0.05

Month	CW2 effluent concentration (mean ± SD)					
	TSS	COD	TN	NH_4_^–^N	TP	PO_4_^3−^
Jan	50.5 ± 0.5 (56.6)	64.1 ± 7.2 (67.8)	21 ± 1.8 (59.2)	9.1 ± 0.9 (67.8)	4.9 ± 0.5 (72.9)	3 ± 0.6 (73.5)
Feb	35.8 ± 1.8 (59.7)	51 ± 5 (68.5)	14.8 ± 2.2 (58.8)	5.4 ± 0.7 (74.4)	5 ± 0.4 (66)	2.8 ± 0.6 (67.8)
Mar	16.8 ± 2.6 (67.1)	17.4 ± 4 (76.3)	6.8 ± 1 (62.8)	1.8 ± 0.3 (81.3)	4.7 ± 0.7 (62.4)	1.9 ± 0.1 (72.1)
Apr	36.9 ± 2 (62)	43.9 ± 3.3 (61.6)	5.3 ± 0.6 (64.9)	1.1 ± 0.1 (82.8)	4.8 ± 0.9 (75.9)	3.2 ± 0.9 (76.4)
May	13.9 ± 2.7 (73.9)	14 ± 3.4 (75.7)	11.2 ± 1.1 (57.4)	3.1 ± 0.8 (76.9)	14.3 ± 1.2 (52.3)	7.4 ± 1 (55.7)
Jun	18 ± 0.9 (70.5)	29.6 ± 1.5 (66.9)	11.9 ± 1.1 (58.8)	4 ± 1.3 (77)	4.3 ± 0.8 (71.7)	2.9 ± 0.5 (72.4)
Jul	5.3 ± 0.3 (83.1)	7.4 ± 1.3 (75.6)	3.4 ± 0.3 (74.2)	0.9 ± 0.1 (73.5)	2.5 ± 0.9 (80.2)	1.2 ± 0.1 (82.9)
Aug	10.4 ± 0.4 (70.8)	15.2 ± 4.7 (78.2)	2.8 ± 0.3 (74.5)	0.7 ± 0.1 (75.9)	2.7 ± 0.3 (74.3)	1.4 ± 0.05 (75)
Sept	37.5 ± 3.3 (59.9)	38.2 ± 2.5 (64.8)	5.6 ± 0.6 (60.3)	0.8 ± 0.1 (84)	16.3 ± 3.9 (42.8)	9.6 ± 0.3 (50.5)
Oct	22.8 ± 1.9 (65.5)	42.8 ± 1.1 (66.5)	7.4 ± 3 (65.1)	3.2 ± 1 (77.5)	9.4 ± 1.6 (36.5)	5.7 ± 0.2 (47.7)
Nov	18.8 ± 1.3 (65.7)	29.7 ± 3.4 (67.8)	4.8 ± 0.9 (64.2)	1.2 ± 0.9 (83.3)	8.7 ± 0.6 (25)	4.8 ± 0.2 (41.5)
Dec	25 ± 1.9 (64)	39.2 ± 3.4 (68)	12.3 ± 1.3 (57.9)	3.7 ± 1.5 (77.2)	13.3 ± 2.4 (32.5)	9.3 ± 0.9 (36.7)
*p*-value	< 0.05	< 0.05	< 0.05	< 0.05	< 0.05	< 0.05
EEPA^*^	< 50	< 250	< 40	< 20		< 5

^*^Ethiopia environmental protection authority, EEPA (2003) discharge standards (mgL^−1^)

**Fig. 6:**
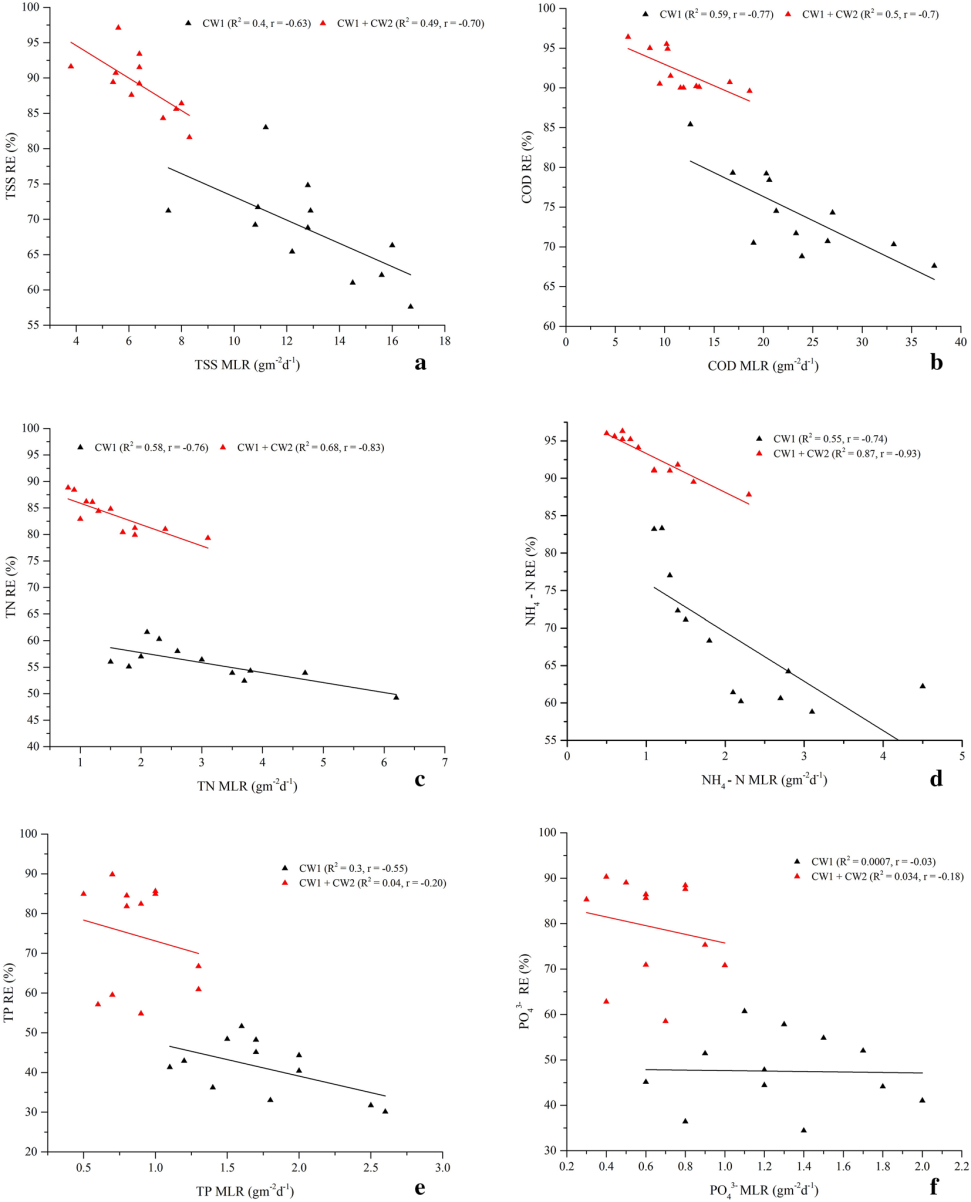
Relationships of MLR and RE of brewery pollutants in the series-connected two-stage HSSFCWs

Overall, the complete system showed an average removal efficiencies of 89 ± 4.1%TSS and 92 ± 2.6%COD. However, significantly varied TSS and COD removals were observed throughout the study period may be due to the influent pollutant loading and seasonal variations. Da Motta Marques et al. (2001) were noticed a very high significant TSS and COD removal variations due to influent loading effect, in which as with loading increased, TSS and COD removals were decreased. Another study conducted by Chang et al. (2007) were also observed a negative response between the COD mass loading rate and removal efficiency. With regard to the present study, the TSS and COD pollutant loading rate fluctuation influences their removal efficiencies (Fig. 6ab). For instance, when the applied influent loading rate of TSS and COD were 8.3 gTSSm^−2^d^−1^ and 18.6 gCODm^−2^d^−1^, the complete system showed 81.6% TSS and 89.6% COD minimal removal efficiencies in January. While the system achieved maximum removal of 97.1% TSS and 96.4%COD when the system loaded with relatively minimum loading of 5.6 gTSSm^−2^d^−1^ and 6.3 gCOD m^−2^d^−1^ in July. Overall, Fig. 6ab described, as the MLR increased, the TSS and COD removal efficiencies were decreased may be due to loss of suspended solids and organic matters without enough contact. Many studies used single stage HSSFCW systems for post-treatment of anaerobically treated sewage wastewaters (Table 3). De Sousa et al. (2003) were examined the efficiency of a *Juncus spp.* planted HSSFCWs for polishing UASB reactor sewage wastewater, and reported 70% to 71%TSS and 79% to 86%COD removals at HRT of 10 days and MLR of 6.64 gCODm^−2^d^−1^. Another study by Von Sperling (2015) was also obtained improved TSS and COD removal of 87.8% and 84.5% respectively using a *T. latifolia* planted HSSFCWs during treatment of UASB reactor sewage wastewater. As compared to this single stage polishing techniques, the present two-stage HSSFCWs exhibits the superior performance. The system also brought higher treatment efficiency as compared to Cheng et al. (2010) reported results of 79.4% of TSS and 75.9%COD removals obtained using a *P. australis* and *P. stratiotes* planted two-stage HSSFCWs for polishing of UASB reactor treated mixture of sewage and swine wastewater at HRT of 13.5 days. These marked removal efficiencies may be associated with the concerted action of combined macrophytes through adhering more solid particles, biodegradation, and consumption of organic matters via the action of consortia of anaerobic and aerobic microorganisms located close to the rhizosphere of the macrophytes and in the pores of the substrate (Theophile et al. 2011; Sa’at et al. 2017; La Bella et al. 2016).

**Table 3:** Comparison performance efficiency of a series connected two-stage HSSFCWs with other research findings

System	Macrophyte used	HRT (day)	MLR (gm^−2^d^−1^) in HF SSCW unit (s)					HF SSCW removal efficiency (%)					References
			COD	TSS	TN	NH_4_–N	TP	COD	TSS	TN	NH_4_–N	TP	
UASB + HF SSCW	*Juncus spp.*	10	6.64	–	1.98	–	0.25	79–86	70–71	63–79	–	9–90	De Sousa et al. (2003)
ABR + HF SSCW	*Z.bonorrienss & T. subalata*	1.5	–	–	4.5	–	1.1	71.4	86.1	90.4	95.8	93.3	Da Motta Marques et al. (2001)
UASB + 2HF SSCW	*Reed* + *Lettuce*	13.7	0.52	1.91	–	0.24	0.1	75.9	79.4	–	96.3	75.1	Cheng et al. (2010)
UASB + HF SSCW	*T. latifolia*	3	14.4	5.8	3.9	3.8	0.3	71	93.7	74.8	88.5	33	El-Khateeb and El-Bahrawy (2013)
UASB + HF SSCW	*T. latifolia*	1.1	–	–	–	–	–	84.5	87.8	–	–	–	Von Sperling (2015)
UASB + 2HF SSCW	*C. aternifoius* + *T. latifolia*	4	11.7	6.4	1.6	1.1	0.9	92	89	83.6	92.9	74.4	This study

### TN and NH_4_– N removal

Phytoremediation of nutrient rich UASB effluent using two-stage HSSFCWs showed promising TN and NH_4_– N removal efficiencies across the stages. From the data, it is apparent that the CW1 removed 55.7 ± 3.4%TN and 65.5 ± 8.8%NH_4_– N. While further polishing with CW2 unit drastically decreased the CW1 effluent, and achieved mean removal efficiencies of 63.2 ± 5.9%TN and 77.6 ± 4.7%NH_4_–N. Moreover, the complete system achieved an improved average removal efficiencies of 83.6 ± 3.3%TN and 92.9 ± 2.9%NH_4_– N (Fig. 4cd and Table 2), and met the national discharge standard limits of 40 and 20 mgL^−1^,respectively, may be due to the combined effect of both macrophytes. Previous study by Sa’at et al. (2017) indicated that *C. alternifolius* based treatment of aerobic palm oil mill effluent at 11 days HRT removed 92% NH_4_^–^N. another study report by Leto et al. (2013) also showed that *C. alternifolius* based treatment of domestic wastewater removed 65.2% TN, and 66.7% NH_4_^–^N. Likewise, the promising removal of TN and NH_4_–N by CW1 may be due to the relatively high DO concentration that has more positive linear relationships with these pollutant outlet concentrations (*R*^2^ = 0.83; *r* = 0.91 for TN, and *R*^2^ = 0.84, *r* = 0.91 for NH_4_^–^N) (Fig. 5b), and agreed the finding of La Bella et al. (2016), who observed the vital role of *C. alternifolius* in liable oxygen transport and enables better nitrification due to its larger root mass, deeper root growth and higher aboveground biomass, and good nitrogen absorption ability (Wijaya et al. 2016). Whereas, in the CW2, a weak relationship was found between DO and these pollutants effluent concentrations (*R*^2^ = 0.65; *r* = 0.81 for TN, and *R*^2^ = 0.64, *r* = 0.81 for NH_4_^–^N) (Fig. 5c), implies that it has limited DO transfer ability, and prevails more denitrification process for improved TN and NH_4_–N removals (La Bella et al. 2016). In another study, Bonanno and Cirelli (2017) also reported similar idea that *T. latifolia* has short root growth, and favors denitrification (Fahlgren 2017).

**Fig. 7:**
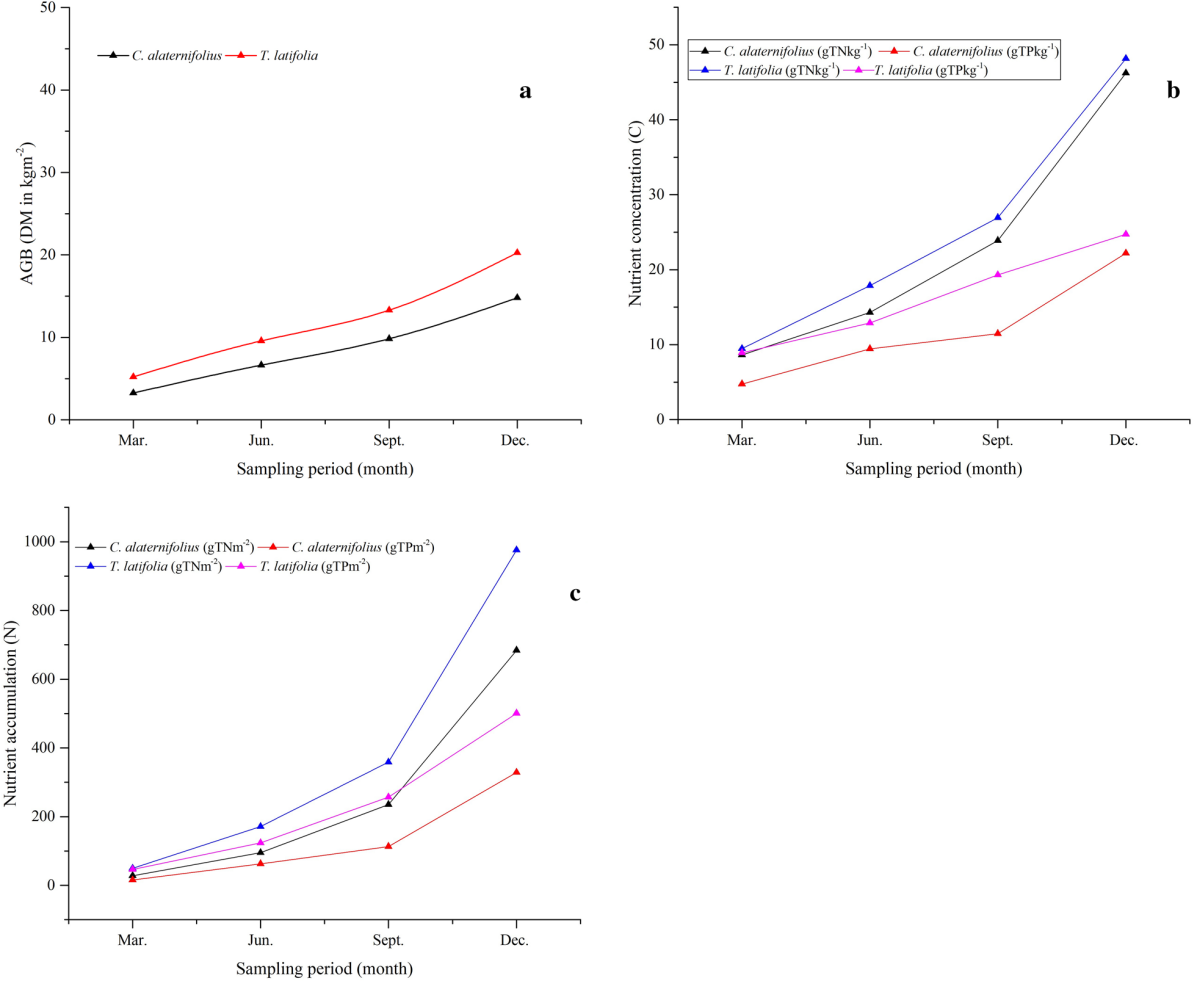
*C. alternifolius* and *T. latifolia* (**a**) aboveground biomass, (**b**) nutrient concentration, and (**c**) Nutrient accumulation

**Fig. 8:**
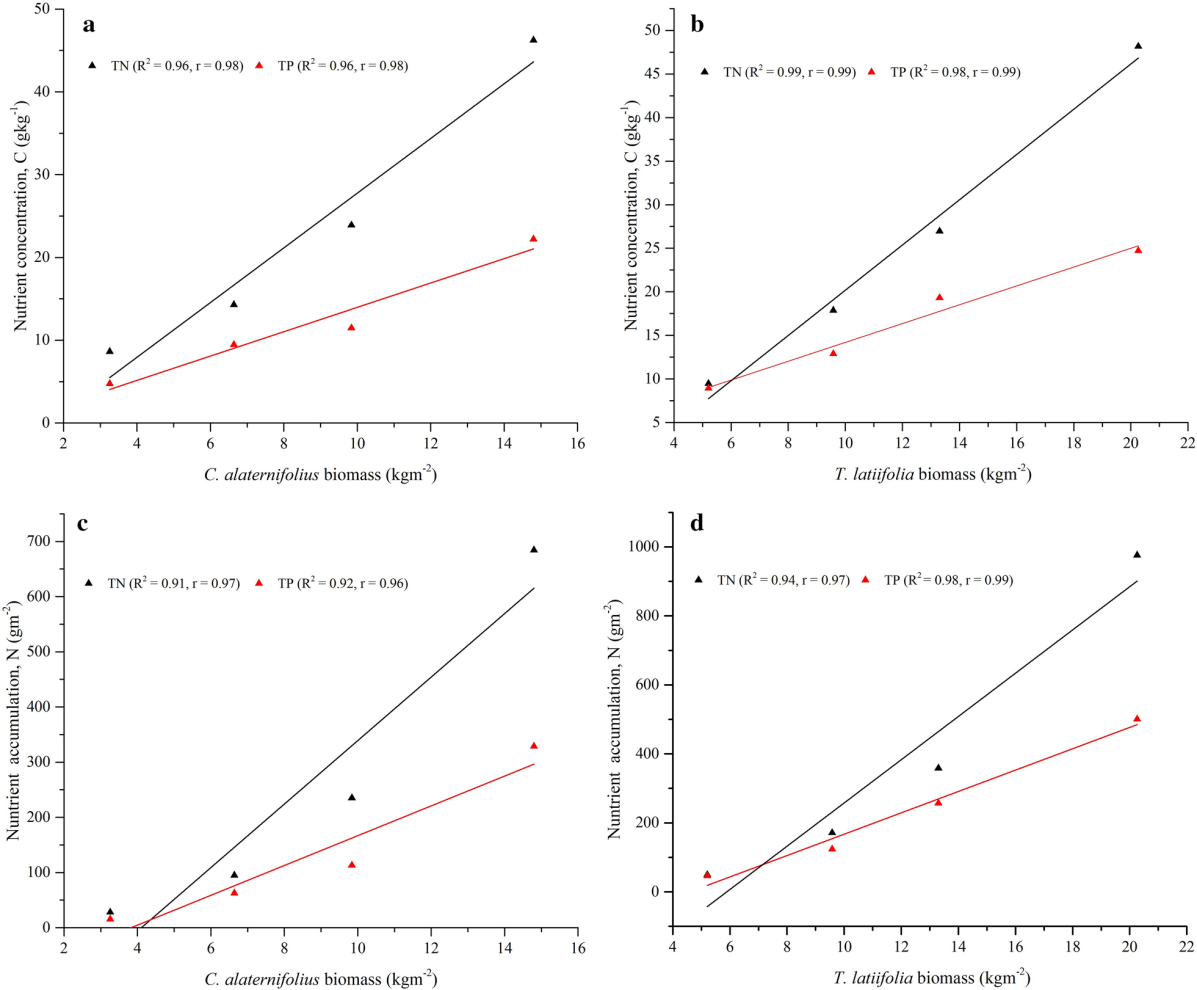
Correlation of (**a**) *C. aternifolius* DM with C, (**b**) *T. latifolia* DM with C, (**c**) *C. aternifolius* DM with N, and (**d**) *T. latifolia* DM with N

Nitrogen and ammonia removal efficiencies by the complete system was variable with the greatest amount of 88.8 ± 1.2%TN and 96 ± 0.2%NH_4_^–^N removal obtained in August at relatively lower loading rate of 0.8 gTNm^−2^d^−1^ and 0.6 gNH_4_^–^Nm^−2^d^−1^. Whereas, the lowest 79.3 ± 2.4% TN and 87.8 ± 1.4% NH_4_ –N removal efficiencies were achieved in January at relatively higher loading rate of 3.1 gTNm^−2^d^−1^ and 2.3 gNH_4_—Nm^−2^d^−1^ (Fig. 6cd). Similarly, Gaballah et al. (2020) and Da Motta Marques et al. (2001) were reported a very high TN and NH_4_–N removal variability due to significant influent loading variations. El-Khateeb and El-Bahrawy (2013) was reported 74.8%TN and 88.5%NH_4_–N removals using a *T. latifolia* planted HSSFCW unit during the polishing of anaerobic reactor treated domestic wastewater at 3 days HRT and loading rate of 3.9 gTNm^−2^d^−1^and 3.8 g NH_4_–N m^−2^d^−1^. However, 83.6% TN and 92.9% NH_4_–N greater removal results were obtained in the present study at loading rate of 1.6 gTNm^−2^d^−1^ and 1.1 gNH_4_–Nm^−2^d^−1^ as compared to many single stage HSSFCW polishing systems mentioned in Table 3 except Da Motta Marques et al. (2001) reported comparable results of 90.4%TN and 95% NH_4_^–^N removal at 1.5 days HRT and loading rate of 4.5 gTNm^−2^d^−1^ using a *Z. bonorriensis* and *T. subalata* planted HSSFCW units during the polishing of anaerobic baffled (ABR) reactor municipal wastewater. Cheng et al. (2010) have also reported similar result of 96.3%NH_4_–N removal obtained using a *P. australis* and *P. stratiotes planted t*wo-stage HSSFCW system during polishing of the UASB reactor treated mixture of sewage and swine wastewater at HRT of 13.5 days and loading rate of 4.5 g NH_4_–N m^−2^d^−1^. In CWs, enhanced removal of nitrogen is performed by volatilization, ammonification, nitrification, denitrification, plant uptake, and matrix adsorption (UN–HABITAT 2008; Saeed and Sun 2012). Macrophytes nutrient uptake play a significant role in the reduction of nutrients; for instance, Wijaya et al. (2016) indicated *C. alternifolius* and *T. latifolia* nitrogen uptake were 0.3 gTNm^−2^d^−1^ and 0.27 gTNm^−2^d^−1^ respectively. But, in this study, measurement of DM of *C. alternifolius* and *T. latifolia* were varied between 3.26 and 14.8 kgm^−2^, and 5.21 to 20.26 kgm^−2^, respectively, (Fig. 7a) with an increased TN concentration variations in between 8.62 and 46.23 gTNkg^−1^ and 9.46 to 48.16 gTNkg^−1^ respectively by *C. alternifolius* and *T. latifolia* (Fig. 7b). Overall, two-stage HSSFCW phytoremediation process showed enhanced nutrient accumulations varied from 28.1 to 684.2 gTNm^−2^ and 49.3 to 975.7 gTNm^−2^,respectively, *C. alternifolius* and *T. latifolia* (Fig. 7c). Strong linearity and Pearson correlations were observed between DM and TN concentration (*R*^2^ = 0.96, *r* = 0.98 for *C. alternifolius*; *R*^2^ = 0.99, *r* = 0.99 for *T. latifolia*) (Fig. 8ab), and between DM and TN accumulation (*R*^2^ = 0.91, *r* = 0.97 for *C. alternifolius*; *R*^2^ = 0.94, *r* = 0.97 for *T. latifolia*) (Fig. 8cd). Generally, more than threefold reductions and consistent decline in TN and NH_4_^–^N concentrations were achieved by the complete system may be due to the concerted action of the combined macrophytes through physicochemical and biological processes, and argued with Zhu et al. (2014), who suggested two-stage CW would provide better nitrogen removal.

### TP and PO_4_^3−^ removal

Post-treatment of phosphorus-rich UASB reactor effluent using CW1 resulted mean removal efficiency 41.1 ± 7%TP and 48.1 ± 7.5%PO_4_^3−^, respectively. Further polishing with CW2 improved the TP and the PO_4_^3−^ mean removal efficiencies of 57.7 ± 19.2%TP and 61.9 ± 16.5% PO_4_^3−^. Further enhanced TP and PO_4_^3−^ removal efficiencies of 74.4 ± 13.3% and 79.5 ± 11.3% were, respectively, achieved by the complete system (Fig. 4ef, Table 2). Overall, significant differences in the removal of TP and PO_4_^3−^ were observed between sampling periods for the complete system, with maximum TP removal (> 80%) from January to August at loading rate varied from 0.5 to 0.9 gm^−2^d^−1^ and > 85% PO_4_^3−^ removal at loading rate varied from 0.3 to 0.7 gm^−2^d^−1^. Also, Gaballah et al. (2020) were also reported that phosphorous removal in a CW system is influenced by loading rate, and conclude that lower loading increase TP removal in CWs. However, in this study, no correlation was found between loading rate and RE of TP and PO_4_^3−^ (Fig. 6ef). De Sousa et al. (2003) were reported 90%TP removal using a *Juncus sp.* planted HSSFCW unit during polishing of anaerobic reactor sewage wastewater at HRT of 10 days and loading rate of 0.25 gTPm^−2^d^−1^. Result comparison of the present study phosphorous removal by the two-stage HSSFCWs was lower than Da Motta Marques et al. (2001) finding, who reported 93.3% of phosphorous removal at 1.5 days HRT and loading rate of 1.1 gPm^−2^d^−1^ using a *Z. bonorriensis* and *T. subalata* planted HSSFCW units during polishing of UASB reactor municipal wastewater, and higher than Cheng et al. (2010) reported result of 75.1%TP removal using a *P. australis* and *P. stratiotes planted t*wo-stage HSSFCWs during polishing of the UASB reactor mixture of sewage and swine wastewater at HRT of 13.5 days and loading rate of 0.1 gPm^−2^d^−1^ (Table 3). In general, two-stage phytoremediation process showed a promising TP and PO_4_^3−^ reduction, which met the national discharge standard limit until August except May. However, after August, almost all, it exceeded the national limit probably due to the saturation of clay media active sites, which decreases the absorption abilities (Ciria et al. 2005). Several studies reported that phosphorous removal in a CW system is dependent on the type of media and macrophytes used. Since these wetland components play a significant role in its removal via media absorption, microbial uptake as well as plant uptake (Badejo et al. 2014). Likewise, Ciria et al. (2005) reviewed that the clay media was the main phosphorous sink and increased its removal efficiency by more than 36% through adsorption. In another case, the presence of macrophytes were achieved over 60% of phosphorus removal (Zamora et al. 2019). In this study, analysis of the TP concentration in *C. alternifolius* and *T. latifolia* was varied from 4.74 to 22.2 gTPkg^−1^ and 8.92 to 24.71 gTPkg^−1^, respectively (Fig. 7b). Application of these macrophytes in the two-stage HSSFCWs contributed an important role in the reduction of phosphorous through uptake of 129.9 and 231.8 gTPm^−2^ by *C. alternifolius* and *T. latifolia*, respectively (Fig. 7c), with cumulative uptake of 708.7 gTPm^−2^. Overall, strong linearity and Pearson correlations were observed between DM and TP concentration (R^2^ = 0.96, r = 0.98 for *C. alternifolius*; *R*^2^ = 0.98, *r* = 0.99 for *T. latifolia*) (Fig. 8ab), and DM and TP accumulation (*R*^2^ = 0.92, *r* = 0.96 for *C. alternifolius*; *R*^2^ = 0.98, *r* = 0.99 for *T. latifolia*) (Fig. 8cd). Wijaya et al. (2016) and Xu et al. (2017) were also observed good phosphorous uptake abilities of *C. alternifolius* and *T. latifolia*. In general, a major phosphorous removal mechanism in a CWs are adsorption, precipitation, storage, plant uptake and biotic assimilation (UN–HABITAT 2008).

## Conclusion

Anaerobic digestion is an ideal sustainable pretreatment option for the treatment of high-strength food processing wastewaters through generating value-added products such as methane and organic fertilizer. Treatment potential evaluation of Kombolcha UASB reactor treatment plant showed 79.3%COD removal with a biogas yield of 1170.1 Nm^3^d^−1^. However, its effluent residual organics, suspended solids and nutrient concentrations exceeded the national discharge standard limit. A system with a combination of macrophytes in the series connected two-stage HSSFCWs has been found suitable for the post-treatment of anaerobically treated brewery effluent. This system showed an enhanced pollutant removal efficiencies of 89%, 92%, 83.6%, 92.9%, 74.4%, and 79.5%, respectively, for TSS, COD, TN, NH_4_–N, TP, and PO_4_^3−^, and meets the tolerable national discharge limit, except for phosphorous. Phosphorous removal was also promising for the initial seven-month operations except May, while in the latter operating periods, almost all it exceeded the discharge limit. Overall, the two-stage HSSFCWs planted with *C. alternifolius* followed by *T. latifolia* is recommended for the post-treatment of the UASB reactor brewery effluent. The study proved that the use of CW as a post-treatment can turn food processing industrial wastes into clean water use for agriculture purposes.

## Data Availability

The data and materials used in this manuscript are available from the corresponding author on request.

## Abbreviations

EEPA: Ethiopian environmental protection authority
APHA: American public health association
HSSFCW: Horizontal subsurface flow constructed wetland
UASB: Up flow anaerobic sludge blanket
HRT: Hydraulic retention time
CW: Constructed wetland
L: Wetland length
W: Wetland width
D: Wetland depth
A_s_: Wetland surface area
p: Media porosity
K_BOD_: Biological oxygen demand rate constant
Q: Hydraulic flow rate
C_i_: Influent concentration
C_e_: Effluent concentration
RE: Removal efficiency
MLR: Mass loading rate
TSS: Total suspended solids
COD: Chemical oxygen demand
BOD: Biological oxygen demand
TN: Total nitrogen
NH_4_–N: Ammonia–nitrogen
TP: Total phosphorous
PO_4_^3^^−^: Orthophosphate
DO: Dissolved oxygen
AGB: Aboveground biomass
DM: Dry weight biomass
